# Screening and analysis of PoAkirin1 and two related genes in response to immunological stimulants in the Japanese flounder (*Paralichthys olivaceus*)

**DOI:** 10.1186/1471-2199-14-10

**Published:** 2013-05-07

**Authors:** Chang-Geng Yang, Xian-Li Wang, Bo Zhang, Bing Sun, Shan-Shan Liu, Song-Lin Chen

**Affiliations:** 1 Yellow Sea Fisheries Research Institute, Chinese Academy of Fisheries Sciences, Qingdao 266071, China; 2 Yangtze River Fisheries Research Institute, Chinese Academy of Fishery Sciences, Wuhan 430223, China; 3 Translational Center for Stem Cell Research, Tongji Hospital, Stem Cell Research Center, Tongji University School of Medicine, Shanghai 200065, China; 4 Bohai Sea Fisheries Research Institute of Tianjin, Tianjin, China

**Keywords:** Akirin, Japanese flounder, NF-κB, Yeast two-hybrid assay, Immunity, HEPN, C1q

## Abstract

A member of the NF-κB signaling pathway, PoAkirin1, was cloned from a full-length cDNA library of Japanese flounder (*Paralichthys olivaceus*). The full-length cDNA comprises a 5′UTR of 202 bp, an open reading frame of 564 bp encoding a 187-amino-acid polypeptide and a 521-bp 3′UTR with a poly (A) tail. The putative protein has a predicted molecular mass of 21 kDa and an isoelectric point (pI) of 9.22. Amino acid sequence alignments showed that PoAkirin1 was 99% identical to the *Scophthalmus maximus* Akirin protein (ADK27484). Yeast two-hybrid assays identified two proteins that interact with PoAkirin1: PoHEPN and PoC1q. The cDNA sequences of *PoHEPN* and *PoC1q* are 672 bp and 528 bp, respectively. Real-time quantitative reverse-transcriptase polymerase chain reaction analysis showed that bacteria could induce the expressions of PoAkirin1, PoHEPN and PoC1q. However, the responses of PoHEPN and PoC1q to the bacterial challenge were slower than that of PoAkirin1. To further study the function of PoAkirin1, recombinant PoAkirin1 and PoHEPN were expressed in *Escherichia coli* and would be used to verify the PoAkirin1*-*PoHEPN binding activity. These results identified two proteins that potentially interact with PoAkirin1 and that bacteria could induce their expression.

## Background

Biological processes are primarily performed and controlled by proteins. Therefore, clarifying the biological functions of proteins and their biological response mechanisms at the cellular level has become the main objective of proteomics research. Protein-protein interactions play a crucial role in various biological functions, including the formation of polymer structure, cell signal transduction, gene regulation, and metabolic pathways. In the post-genome sequencing era, protein interaction bridges the gap between prediction of the relationship between the proteins and the annotation of important genes. Thus, comprehensive analysis of protein-protein interactions is crucial for the full understanding of proteomics [1]. Studies of protein-protein interactions not only can reveal the protein function on the molecular level but also are critical for understanding growth, development, differentiation and apoptosis and other crucial life activities, such studies also provide an important theoretical basis for disease mechanisms, disease treatment, disease prevention and drug development. The yeast two-hybrid system is a simple, but powerful, tool for detecting interactions between proteins and has been widely applied in many research areas. Recently, the yeast two-hybrid system has been used to study the large-scale interaction group in viruses, bacteria, Drosophila, and *Caenorhabditis elegans*[2-7].

Nuclear factor κB (NF-κB) is a nuclear transcription factor that plays a key role in the regulation of apoptosis, viral replication, cancer, inflammation and the regulation of the expression of other related genes. In particular, NF-κB can be activated by a variety of stimulatory factors, including cytokines, lymphokines, UV, pharmaceutical preparations, and growth and stress factors. Such activation of NF-κB is part of the stress response. Although many members in the NF-κB signaling pathway have been identified in the past 20 years, a highly conserved protein Akirin, a member in the NF-κB signaling pathway, was recently identified in a study of the immune defense system at 2008 [8]. This 20–25-kDa protein participates in the regulation of gene expression in many physiological processes, including the insect and mammalian innate immune response [8], cancer, insect reproduction and arthropods growth [9,10]. Knockout of the Akirin gene led to embryonic lethality of mice and caused death or reduced growth in Drosophila, ticks, and nematodes. Consequently, Akirin is considered important in animal development [11]. Akirin cannot directly combine with DNA, but interacts with the promoter or assisting factors that inhibit the transcription of genes encoding such proteins as the 14-3-3 protein and the helix-loop-helix transcription factor, Twist [12]. Research on fish Akirin has been limited to the analysis of gene structure and function in several species [13-15]. Furthermore, Akirin’s interaction mechanism requires further investigation.

The Japanese flounder (*Paralichthys olivaceus*) is economically important and is widely cultured in Europe and China. However, flounder diseases have a serious impact on the aquaculture industry. Recently, to explore the molecular mechanisms of disease resistance and host-pathogen interactions in this species, Nam’s team [16,17] constructed a cDNA library from an immune stimulated Japanese flounder. In addition, many immune-related genes including those encoding STAT, Nramp (natural resistance associated macrophage protein), MHCIIA and IIB (major histocompatibility complex class II) have also been investigated [18-20].

As an important protein required for NF-κB-dependent gene regulation in the immune response, little is known about Akirin’s function, interacting proteins, and regulation mechanism. In this study, we screened for Akirin interacting proteins and analyzed the interaction mechanism using the yeast two-hybrid system and a cDNA library of flounder immune tissue. Two possible interacting proteins were identified: PoHEPN (a higher eukaryotes and prokaryotes nucleotide-binding domain (HEPN) protein) and PoC1q (complement component C1, q subunit). The expression profiles of PoAkirin1, PoHEPN and PoC1q were also analyzed by a bacterial challenge test. This study increases our understanding of the Akirin family, and provides a theory of flounder immunity and disease resistance mechanisms.

## Methods

### Ethics statement

The Yellow Sea Fisheries Research Institute’s animal care and use committee waived the need for ethical approval, as this is not required in China.

### Experimental animals

Japanese flounders with an average weight of 200 g were obtained from Haiyang Fisheries Company in Yantai and raised in a breeding tank with seawater (20°C). For cloning and tissue expression analysis, RNA was extracted from 13 tissues (brain, gill, skin, muscle, fin, heart, liver, spleen, eye, pituitary, kidney, head kidney, and intestine) from three individuals. For the bacterial challenge experiment, RNA was extracted from three tissues (liver, spleen, and kidney) from three individuals.

### Bacterial challenge

The bacterium *Vibrio anguillarum*, which is pathogenic in Japanese flounders, was cultured at 28°C to mid-logarithmic growth on 2216E medium, centrifuged to collect the bacteria and suspended in 0.9% saline [21,22]. A cell counter was used to measure the number of bacteria in the suspension. A final concentration of 7 × 10^6^ cfu of *V. anguillarum* was used for each injection, and 0.9% saline was used as the negative control. At 6 h, 12 h, 24 h, 48 h, 72 h and 96 h post-injection, three individuals from each time point were sacrificed and tissues were used for RNA extraction. For the negative control, tissues were taken 12 h after the saline injection.

### RNA extraction

Total RNA was isolated from 500 mg powdered fish tissues by homogenization in 5 mL TRIzol (Invitrogen), held at room temperature for 5 min. An aliquot of chloroform (1 mL) was added to each extract, and the resulting mixture was centrifuged (10 min, 13,000 g). The aqueous layer was transferred to a clean tube, and the RNA firstly precipitated by the addition of 3 mL isopropanol, and then pelleted by centrifugation (15 min, 13,000 g). The RNA pellet was washed twice with 75% ethanol and re-suspended in diethylpyrocarbonate-treated water. After DNA removal (Turbo DNA-free kit, Ambion), RNA integrity was detected using agarose gel electrophoresis, and the concentration of RNA was quantified spectrophotometrically.

### Cloning of PoAkirin1

Based on the turbot Akirin1 gene sequence, primers (AKI-R-S1 and AKI-R-A1) were designed to amplify a conservative fragment. The 5′ and 3′ fragments of PoAkirin1 gene were amplified from the flounder full-length cDNA Library (a mix of liver, spleen and kidney).

### Sequence analysis

Translation was performed using DNASTAR software. The conserved domain analysis and BLAST analysis was performed at http://blast.ncbi.nlm.nih.gov/Blast.cgi, containing blastn, blastp and tblastp. The PSORT II server (http://psort.ims.u-tokyo.ac.jp) was used to predict the putative nuclear localization signal (NLS). The alignment of Akirins from different species was performed using the ClustalW alignment program, and the phylogenetic tree was constructed on the basis of the proportion of the amino acid differences (p-distances) determined by the neighbor-joining method [23] using MEGA 3 software [24]. The following proteins were used in the alignment: AAF50569 [*Drosophila melanogaster*], AAN12062 [*D. melanogaster*], ADK27484 [*Scophthalmus maximus*], BAI49701 [*Marsupenaeus japonicus*], ADK26453 [*Gallus gallus*], NP_001161992 [*Salmo salar*], ACV49724 [*Oncorhynchus mykiss*], ACV49723 [*O. mykiss*], ACV49722 [*O. mykiss*], ACV49721 [*O. mykiss*], ACV49720 [*O. mykiss*], ACV49719 [*O. mykiss*], ACV49718 [*O. mykiss*], ACV49717 [*O. mykiss*], ACV49716 [*Salmo trutta*], ACV49715 [*S. trutta*], ACV49714 [*S. trutta*], ACV49712 [*S. trutta*], ACV49710 [*S. trutta*], ACV49708 [*S. salar*], ACV49706 [*S. salar*], ACV49704 [*S. salar*], ACV49702 [*S. salar*], ACV49700 [*Salvelinus alpinus*], ACV49697 [*S. alpinus*], ACV49696 [*S. alpinus*], ADK39312 [*Caligus rogercresseyi*], NP_001107272 [*Danio rerio*], NP_001007187 [*D. rerio*], NP_988914 [*Xenopus (Silurana) tropicalis*], NP_001085484 [*Xenopus laevis*], NP_001025225 [*Rattus norvegicus*], DAA31047 [*Bos taurus*], DAA26175 [*B. taurus*], XP_002715780 [*Oryctolagus cuniculus*], XP_002714617 [*O. cuniculus*], XP_002708555 [*O. cuniculus*], XP_002708554 [*O. cuniculus*], XP_002736520 [*Saccoglossus kowalevskii*], AAH97074 [*D. rerio*], AAH03291 [*Mus musculus*], AAH61612 [*X. tropicalis*], CAM16479 [*M. musculus*], AAI19746 [*Homo sapiens*], AAH05051 [*H. sapiens*], NP_001103557 [*B. taurus*], NP_001016080 [*X. tropicalis*], NP_001035003 [*R. norvegicus*], and AEO17042 [*Ovis aries*].

### Quantitative real-time RT-PCR (RT-qPCR)

The RT-qPCR protocol adhered to the ‘Minimum Information for Publication of Quantitative Real-time PCR experiment guidelines [25]. The relative mRNA steady-state level was measured by RT-qPCR. The total RNA from different tissues was prepared using the TRIzol reagent. The cDNA was synthesized from each RNA sample (500 ng) using a PrimeScript® RT reagent kit (Takara, China), following manufacturer’s protocol. RT-qPCR was conducted on an Applied Biosystems 7500 Real-Time PCR System with SYBR® Premix Ex Taq™ (Takara, China). Genes encoding β-actin (for normal tissue types and infected kidney), a-tubulin (for infected spleen) and glyceraldehyde-3-phosphate dehydrogenase (for infected liver) were selected for normalization, as their expressions have been reported to be stable [26-28]. The RT-qPCR primer pairs were designed to generate a product size of 150–250 bp and a Tm of 60 ±1°C. cDNA from 13 normal and infected tissues was chosen for the detection of PoAkirin1, PoHEPN and PoC1q expression, using the gene-specific primers AKI-R-S1, AKI-R-A1 (for PoAkirin1); HEPN-R-S1, HEPN-R-A1 (for PoHEPN); and C1q-R-S1, C1q-R-A1 (for PoC1q) (Table 1). The primers β-actin-s1, β-actin-a1, GAP-S1, GAP-A1, and TUBA-S1 and TUBA-A1 were used to amplify the β-actin, a-tubulin, and glyceraldehyde-3-phosphate dehydrogenase fragments, respectively (Table 1). PCR efficiency (E) of these primers were between 92 and 110% and correlation coefficient (R2) ranged from 0.991 to 0.998.

**Table 1:** Oligonucleotide primers used in this study

**Name**	**Sequence**	**Purpose used**
AKI-F-S1	ATGGCCTGCGGAGCGACGTT	ORF amplification
AKI-F-A1	TCAGGAGACATAACTAGCAGGCCG	ORF amplification
β-actin-s1	GCTGTGCTGTCCCTGTA	RT-PCR
β-actin-a1	GAGTAGCCACGCTCTGTC	RT-PCR
GAP-S1	CAACGGCGACACTCACTCCTC	RT-PCR
GAP-A1	TCGCAGACACGGTTGCTGTAG	RT-PCR
TUBA-S1	TGACATCACAAACGCCTGCTTC	RT-PCR
TUBA-A1	GCACCACATCTCCACGGTACAG	RT-PCR
AKI-R-S1	AGGACCAGCCCTCGTTCACACT	RT-PCR
AKI-R-A1	TCCGTATCTTCGCATGATCTGGT	RT-PCR
HEPN-R-S1	TACAAGGACAATGGTGGGGG	RT-PCR
HEPN-R-A1	GGCAAGGGCTGAGATGGAG	RT-PCR
C1q-R-S1	CTCCAGAAAACGAAGCAGGC	RT-PCR
C1q-R-A1	TGTCGCACATCATCAAGTGAAC	RT-PCR
AKI-F-A7	GCGTCGACGGAGACATAACTAGCAG	Plasmid construction
AKI-T-A1	GCGGTACCGGAGACATAACTAGCAG	Plasmid construction
AD-AKI-S1	GTGAATTCATGGCCTGCGGAGCGACGTT	Plasmid construction
AD-AKI-A1	CAGCTCGAGTCAGGAGACATAACTAGCAG	Plasmid construction
C1q-S1	GCCGAATTCAAGGGGGCACCAGGTCTTAA	Plasmid construction
C1q-A1	CAGGTCGACTCAGGCCGTGGGGAAGACGA	Plasmid construction
HEPN-S3	GTGAATTCGGCACGAGGCTCAGGTGGCA	Plasmid construction
HEPN-A2	CAGCTCGAGAGCCTCCTCTTTGTTTGGCC	Plasmid construction
HEPN-A3	CAGCTCGAGGGAAGGTTGACCGTGCCTTT	Plasmid construction
HEPN-S5	AGTGAATTCTCTGGCAGCTCCATCTCAGC	Plasmid construction
HEPN-A4	CAGCTCGAGCTATTTCACATATGCCTCAAC	Plasmid construction
HEPN-ET-S1	CCGAATTCAGGCTCAGGTGGCATCCT	Plasmid construction
HEPN-ET-A1	GTGCTCGAGCTATTTCACATATGCCTCA	Plasmid construction

RT-PCR was carried out with a 1 μl cDNA sample, 10 μl SYBR® Premix Ex Taq™, 0.4 μl ROX Reference Dye II, 0.4 μl PCR forward/reverse primers (10 μM) and 7.8 μl nuclease-free water. The thermo-cycling conditions for the reaction were as follows: 95°C for 30 s, followed by 40 cycles of 95°C for 15 s and 61°C for 34 s. The reaction was carried out with triplicate with duplicates of each sample. Data (normalized Ct values) from the treated and control tissues templates were compared, and the 2-ΔΔCT method was selected for relative quantification. All data were expressed as the mean ± S.D. and analyzed by one-way analysis of variance to determine significant differences between samples, using SPSS 16.0. Values were considered statistically significant when P < 0.05 or P < 0.01.

### Plasmid construction

**Figure 1:**
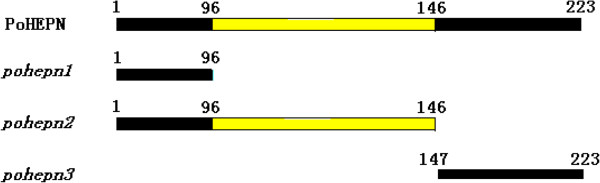
**Sketch map of the *PoHEPN *deleted segment.** PoHEPN: normal *PoHEPN*; *pohepn1*: deletion of 96-223aa; *pohepn2*: deletion of 146-223aa; *pohepn3*: deleted 1-146aa.

Using the sequences of the open reading frames (ORFs) of the PoAkirin1, PoHEPN and PoC1q genes, primers were designed to construct pGEX4T-1-PoAkirin1 (for expression in *E. coli*), pET30a-PoHEPN (for expression in *E. coli*), pGBKT7-PoAkirin1 (for Yeast two-hybrid assay), pGADT7-PoAkirin1 (for Yeast two-hybrid assay), pGBKT7-PoC1q (for Yeast two-hybrid assay), pGADT7-PoHEPN-M1 (for Yeast two-hybrid assay), pGADT7-PoHEPN-M2 (for Yeast two-hybrid assay), and pGADT7-PoHEPN-M3 (for Yeast two-hybrid assay) vectors. The primer sequences and restriction sites corresponding to the vectors are shown in Table 1 and Table 2. The mutation vectors pGADT7-PoHEPN-M1, pGADT7-PoHEPN-M2, and pGADT7-PoHEPN-M3 mutation vectors were constructed according to the model in Figure 1.

**Table 2:** Primer names and restriction sites used to construct the expression vectors

**Name**	**Restriction site**	**Vector**
AKI-F-A7	*Sal*I	pGBKT7-PoAkirin1
AKI-T-A1	*Kpn*I	
AD-AKI-S1	*EcoR*I	pGADT7-PoAkirin1
AD-AKI-A1	*Xho*I	
C1q-S1	*EcoR*I	pGBKT7- PoC1q
C1q-A1	*Sal*I	
HEPN-S3	*EcoR*I	pGADT7- PoHEPN-M1
HEPN-A2	*Xho*I	
HEPN-S3	*EcoR*I	pGADT7- PoHEPN-M2
HEPN-A3	*Xho*I	
HEPN-S5	*EcoR*I	pGADT7- PoHEPN-M3
HEPN-A4	*Xho*I	
HEPN-ET-S1	*EcoR*I	pET30a-PoHEPN
HEPN-ET-A1	*Xho*I	

### Yeast two-hybrid screening and mating assays

The pGBKT7-PoAkirin1 plasmid and DNA plasmids for cDNA library clones were individually transformed into *Saccharomyces cerevisiae* strain Y2HGold and *S. cerevisiae* strain Y187, following the manufacturer's instructions in the Matchmaker™ Gold Yeast Two-Hybrid System User Manual. Approximately 1 × 10^7^ library clones were screened by yeast mating with selection by growth for 3–5 days at 30°C on agar media lacking Leu and Trp, but with X-α-galactosidase and Aureobasidin A.

### Recombinant protein expression in *Escherichia coli*

The recombinant plasmids pGEX4T-1-PoAkirin1, pET30a-PoHEPN, pGEX4T-1, and pET30a were transformed into *E. coli* BL21(DE3) competent cells. After growth at 37°C, 1.0 mM IPTG was added to the transformed cells and incubation continued for 4 h. Sodium dodecyl sulfate polyacrylamide gel electrophoresis (SDS-PAGE) analysis of total proteins was used to detect recombinant protein expression. No IPTG and cells transformed with pGEX-4 T-1 or pET30a and induced with IPTG were used as negative controls.

### Western blotting analysis

The protein concentrations of the samples were determined using an Enhanced BCA Protein Assay Kit (Beyotime Institute of Biotechnology, China). Recombinant PoHEPN and PoAkirin1 were serially diluted (to 5 ng) and separated by 12% SDS-PAGE and transferred to a BioTrace NT Nitrocellulose Transfer Membrane (PALL, USA). The membranes were blocked and then incubated with a 1 μg/mL dilution of the primary antibody (monoclonal antibodies against His or GST (glutathione-S-transferase; Uscn Life Science Inc., Wuhan, China)). After washing, the membranes were incubated with the secondary antibody, horseradish peroxidase-conjugated goat anti-mouse IgG (1:1,000 dilutions) (Beyotime Institute of Biotechnology, China). Reactive proteins were detected using chemiluminescence (ECL Western Blotting Analysis System, Thermo Fisher Scientific Inc., IL, USA).

## Results

### Cloning and sequence analysis of PoAkirin1

**Figure 2:**
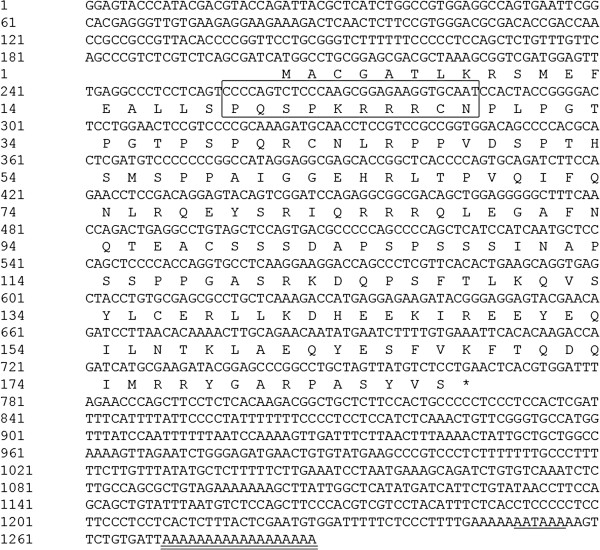
**Nucleotide sequence (above) and deduced amino acid sequence (below) of *PoAkirin1*.** Nucleotides are numbered from the first base at the 5’end. Amino acids, shown as one letter abbreviations, are numbered from the initiating methionine. The predict NLS site is boxed, the AATAAA box is underlined, and the poly(A) region is double-underlined. The stop codon is marked by an asterisk.

**Figure 3:**
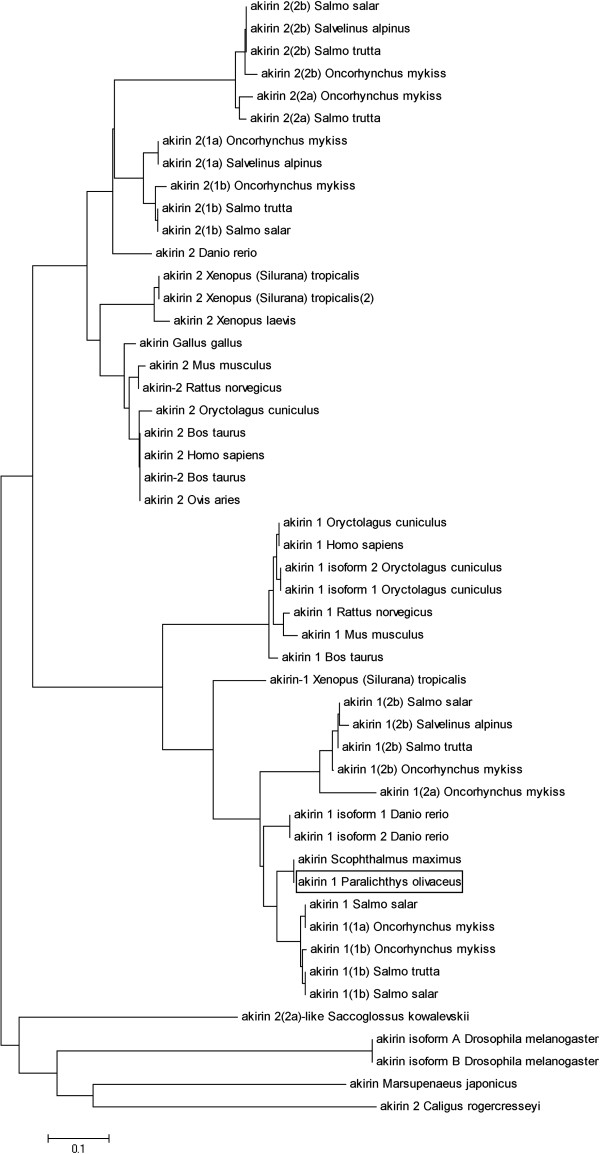
**Phylogenetic tree, constructed by the neighbor-joining algorithm using ClustalW Multiple Alignment, showing the relationship between Akirin proteins.** The PoAkirin1 protein is boxed.

**Figure 4:**
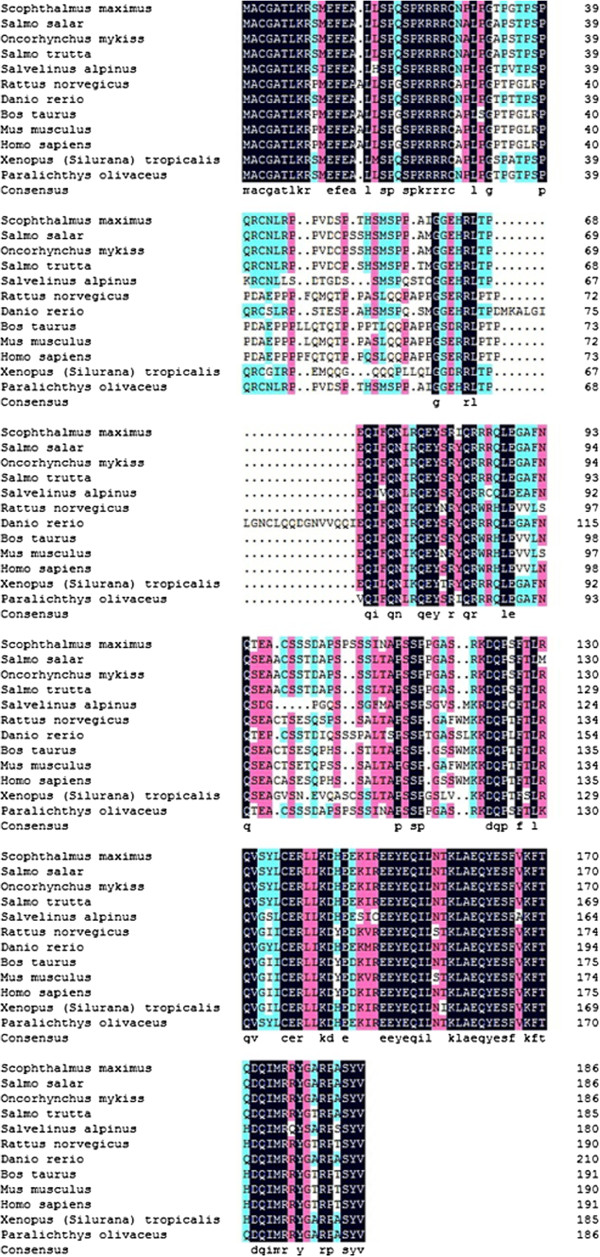
**Alignment of amino acid sequences of PoAkirin1 and other Akirin1 proteins.** Identical residues are shown in white letters with a black background. Sequence accession nos.: *Scophthalmus maximus*, ADK27484.1; *Salmo salar*, NP_001161992; *Oncorhynchus mykiss*, ACV49717; *Salmo trutta*, ACV49710; *Salvelinus alpines*, ACV49696; *Danio rerio*, NP_001025225; *Rattus norvegicus,* NP_001107272; *Bos taurus*, DAA31047; *Mus musculus*, AAH03291; *Homo sapiens*, AAI19746; *Xenopus (Silurana) tropicalis*, NP_001016080; *Oryctolagus cuniculus*, XP_002708554.

The PoAkirin1 cDNA acquired from the full-length cDNA Library of Japanese flounder comprised a 5′UTR of 202 bp, an ORF of 564 bp encoding a polypeptide of amino acids and a 521-bp 3′UTR with a poly (A) tail (Figure 2). Phylogenetic analysis showed that the PoAkirin1 gene clustered together with fish Akirin1 genes (Figure 3, 4).

The PoAkirin1 cDNA encodes a putative protein of 187 amino acids with a predicted molecular mass of 21 kDa and an isoelectric point (pI) of 9.22. A putative NLS was predicted in the N-terminus of the protein (Figure 2). The sequence of PoAkirin1 has been submitted to GenBank with the accession number KC190111.

### Cloning and testing the bait construct for autoactivation

**Figure 5:**
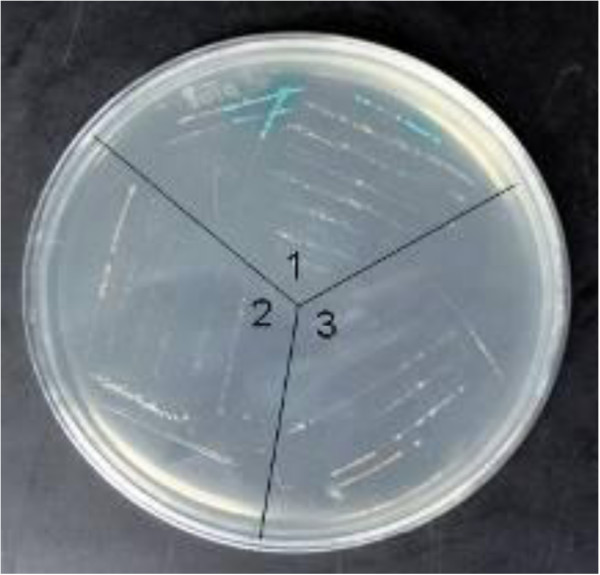
**Transcription activation of PoAkirin1.** The *PoAkirin1* gene was fused to the GAL4 DNA binding domain (GAL4-DB) in the vector pGBKT7. The positive control plasmid was pGBKT7-53. The negative control plasmid was pGBKT7-Lam.

Bait plasmid (100 ng; pGBKT7-PoAkirin1) was transformed into yeast strainY2H Gold and selected on medium SD/Trp (lacking Trp). No autoactivation of fish Akirin1 expression was observed (Figure 5).

### Yeast two-hybrid screening of a Japanese flounder library using bait pGBKT7-PoAkirin1

**Figure 6:**
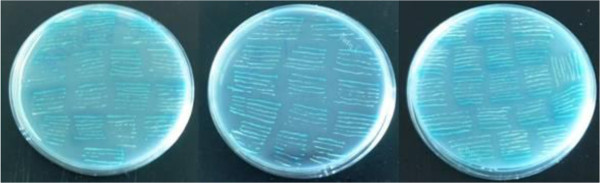
***PoHEPN *cDNA sequence and putative conserved domains.**

**Figure 7:**
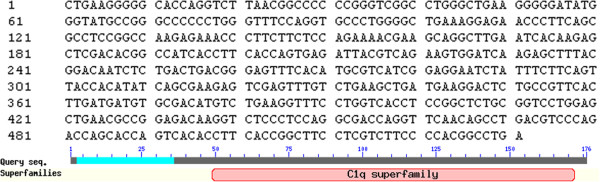
PoCl1q cDNA sequence and putative conserved domain.

**Figure 8:**
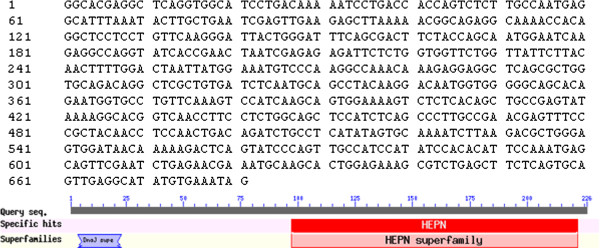
Forty-nine positive clones that may interact with the PoAkirin1 from the screening library.

**Figure 9:**
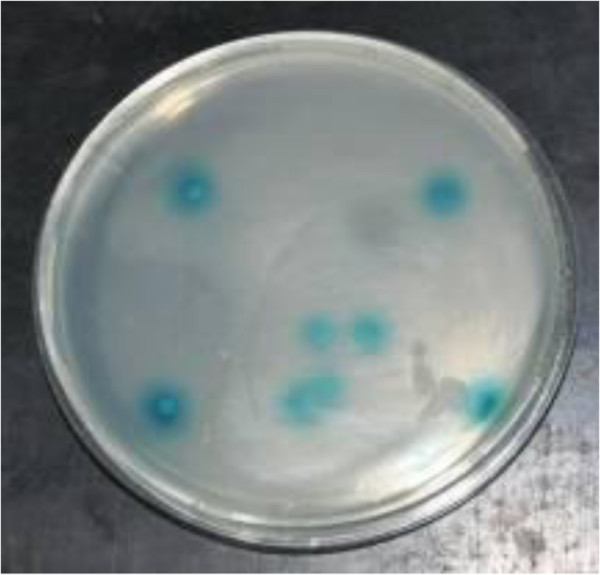
Yeast rotary verify to PoC1q and PoAkirin1.

**Figure 10:**
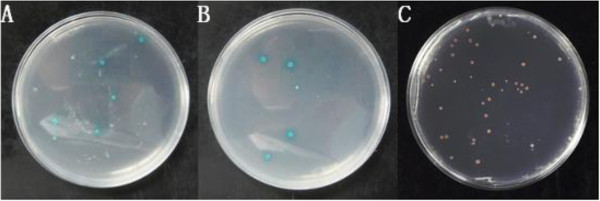
**Growth yeast transformed with the *PoHEPN *deletion segment and PoAkirin1 in SD/–Leu/–Trp/x-α-gal. A**: *pohepn1*; **B**: *pohepn2*; **C**: *pohepn3*.

Using PoAkirin1 as the bait to screen interactions in the library, 49 positive clones were selected. Yeast plasmids extracted from positive clones were transformed into *E. coli* TOP10 and sequenced. DNAMAN was used to analyze the sequence information and BLASTP at the National Center for Biotechnology Information (http://ncbi.nlm.nih.gov/blast) was used to perform the alignments. The results showed that there were seven possible interacting proteins (CD63, HEPN, C1q, T-complex protein 1, voltage-dependent anion channel, asparaginyl endopeptidase and Chaperonin) among the 49 positive clones (Figure 6). To verify the interaction of the proteins, we chose the possible immune-related protein (C1q)   (Figure 7) and the protein predicted in the nucleus (HEPN) (Figure 8) to perform yeast rotary and domain verification tests. The results showed that the yeast grew with a blue color in the yeast rotary verification test using PoC1q and PoAkirin1, and the yeast expressing PoHEPN with a 96–223 aa segment deleted and PoAkirin with a 146–223 aa segment deleted were blue when grown on SD/–Leu/–Trp/x-α-gal. (Figures 1, 9, and 10).

### PoAkirin1 and PoHEPN proteins expression in *E. coli* and western blotting analysis

The recombinant plasmid pGEX-4T-1-Akirin and empty plasmid pGEX-4T-1 were transformed into *E. coli*. After induction by 1 mM IPTG for 4 h, bacterial lysates contained a protein band with a Mr of approximately 47 kDa, as anticipated. The lysates from bacteria transformed with the empty vector pGEX-4 T-1 and that had no IPTG induction did not contain this band (Figures 12A).

**Figure 11:**
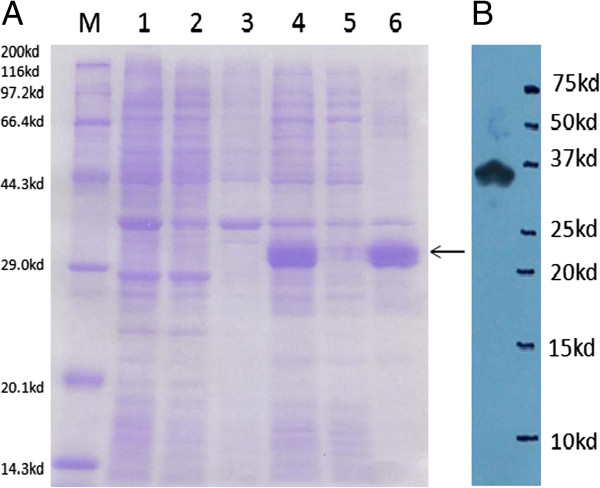
**SDS-PAGE and Western blot analysis of PoHEPN protein. A:** SDS-PAGE analysis of PoHEPN protein expression. M: Protein MW marker (Broad); 1: BL21(DE3)T1R induced whole cell; 2: BL21(DE3)T1R induced Supernatant; 3: BL21(DE3)T1R induced precipitate; 4: pET30-HEPN induced whole cell; 5: pET30-HEPN induced whole cell; 6: pET30-HEPN induced whole cell. **B**: Western blot analysis of PoHEPN protein.

Similarly, after induction by 1 mM IPTG(Isopropyl β-D-1-Thiogalactopyranoside) for 4 h, bacterial lysates with the recombinant plasmid pET30a-HEPN showed a protein band with an Mr of approximately 31 kDa, which corresponded to the predicted size. This band was not present in the negative control bacterial lysates (Figures 11A).

**Figure 12:**
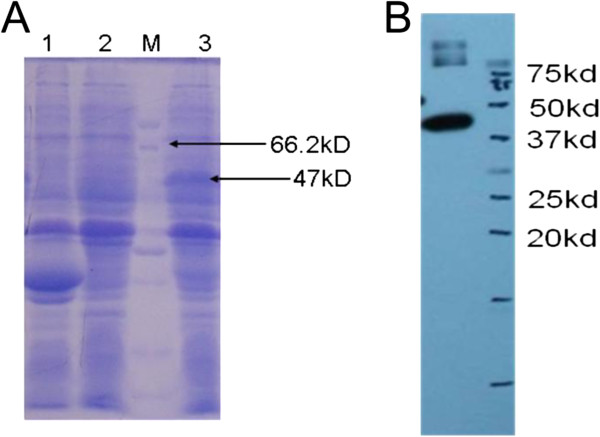
**SDS-PAGE and Western blot analysis of PoAkirin1 protein. A**: SDS-PAGE analysis of PoAkirin1 protein expression. 1: pGEX4T-1 induced; 2: pGEX4T-1- PoAkirin1 non-induced; 3: pGEX4T-1- PoAkirin1 induced; M: Protein MW marker. **B**: Western blot analysis of PoAkirin1.

The recombinant PoHEPN and PoAkirin1 contain His and GST tags; therefore, western blotting was used to detect the expressed proteins using His and GST monoclonal antibodies (Figures 11B, 12B).

### Sequence analysis and expression profile of PoAkirin1, PoHEPN, and Poc1q in various tissues

Yeast two-hybrid screening identified a 672-bp *PoHEPN* cDNA, which encoded a protein with a conserved HEPN domain at the C-terminus. The *PoC1q* gene was 528 bp, and analysis of the predicted protein indicated that it contained a conserved C1q domain at the C-terminus (Figures 7 and 8).

**Figure 13:**
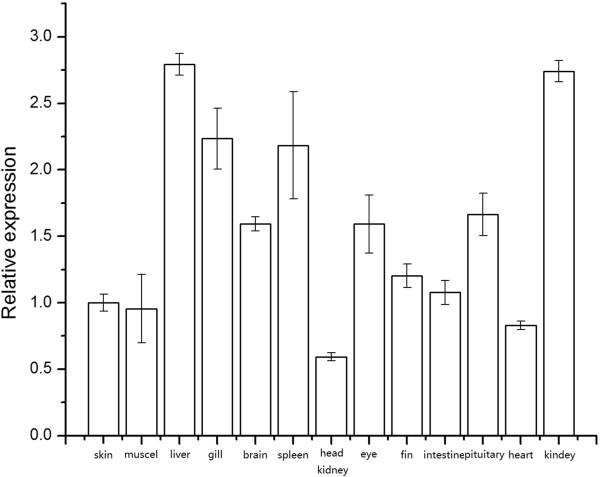
**Expression pattern of *PoAkirin1 *in different tissues of the Japanese flounder detected by quantitative RT-PCR. ***PoAkirin1* mRNA was expressed in all tissues detected. The β-actin gene was used as an internal control to calibrate the cDNA template for all the samples. All data are expressed as the mean ± S.D. (n = 3).

**Figure 14:**
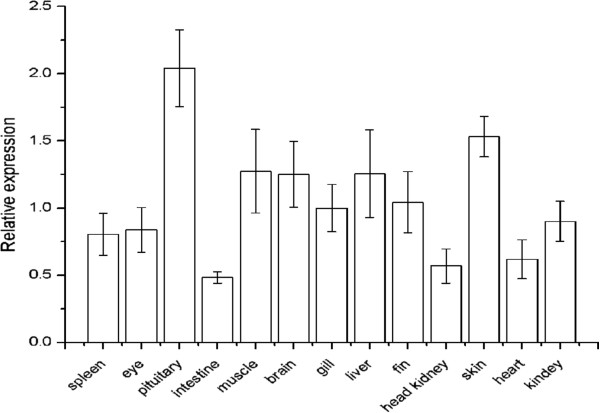
**Expression pattern of *PoHEPN *in different tissues of the Japanese flounder detected by quantitative RT-PCR. ***PoHEPN* mRNA was expressed in all tissues. The β-actin gene was used as an internal control to calibrate the cDNA templates for all samples. Data are expressed as the mean ± S.D. (n = 3).

**Figure 15:**
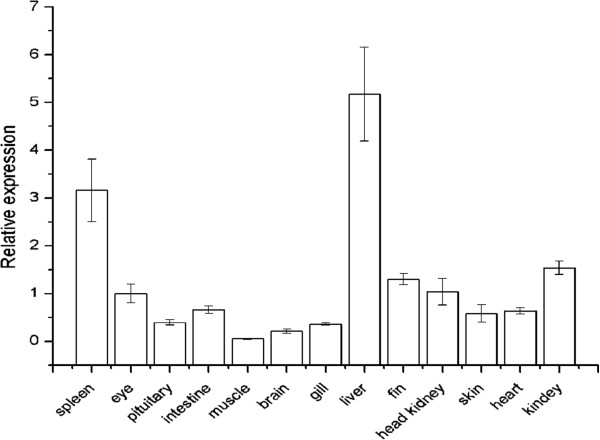
**Expression pattern of *PoC1q *in different tissues of Japanese flounder detected by quantitative RT-PCR. ***PoC1q* was expressed in all tissues, with higher expression levels in the liver. The β-actin gene was used as an internal control to calibrate the cDNA templates for all the samples. Data are expressed as the mean ± S.D. (n = 3).

RT-qPCR was used to quantify the expressions of *PoAkirin1*, *PoHEPN* and *PoC1q* mRNA in different flounder tissues in normal and pathogen-challenged individuals. The mRNA transcripts of *PoAkirin1*, *PoHEPN* and *PoC1q* were detected in all tissues of normal individuals. The *PoAkirin1* mRNA showed the highest expression in liver, and was highly expressed in the spleen and kidney. *PoAkirin1* was also expressed in the other tissues (Figure 13). In most tissues examined, the *PoHEPN* expression levels did not differ significantly, with slightly higher levels in the pituitary and skin. In comparison with other tissues, *PoC1q* expression was relatively high in the brain, gills, pituitary, skin, heart and eyes. In the liver and spleen, the expression of *PoC1q* significantly higher compared with other tissues (p < 0.01). The expression in the liver was 25 and 100 times higher than in the brain and muscle, respectively. (Figures 14 and 15).

**Figure 16:**
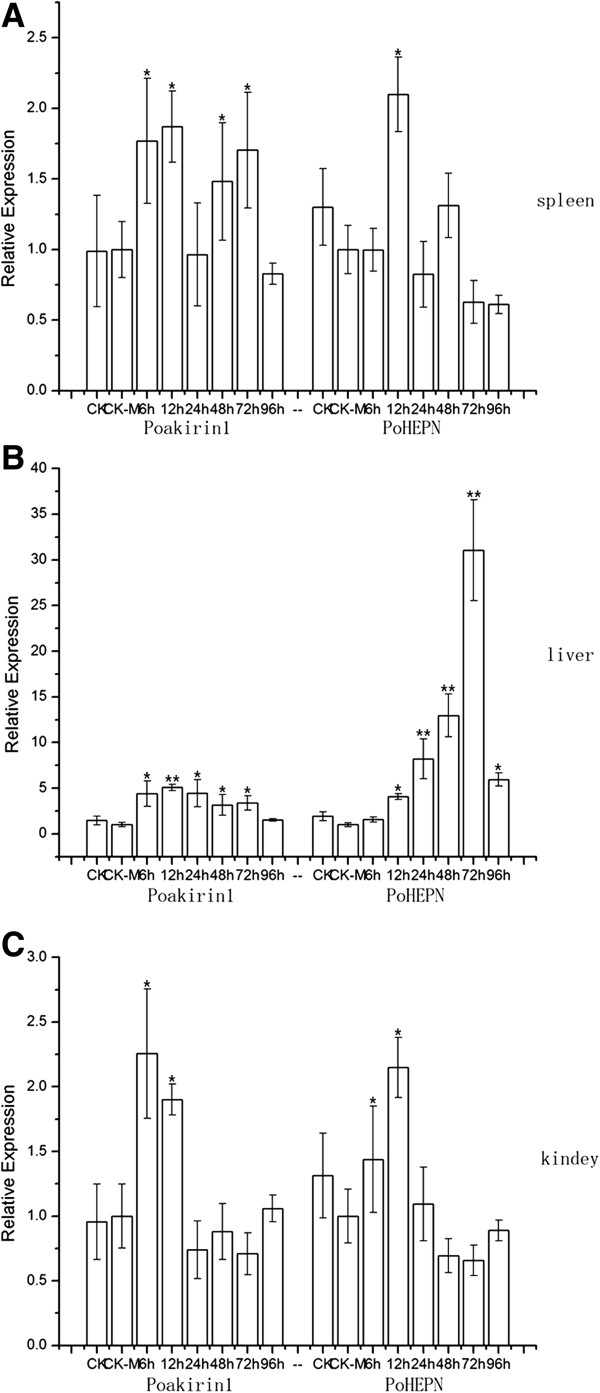
**Quantitative RT-PCR analyses of the expression level of *PoAkirin1 *and *PoHEPN *mRNA in the spleen (A), liver (B), and kidney (C) after injection with *Vibrio anguillarum*.** Figures show the relative expression levels of *PoAkirin1* and *PoHEPN* at 6 h, 12 h, 24 h, 48 h, 72 h and 96 h post-injection. CK-M represents the tissues at 12 h after saline solution injection, and CK means live fish under normal conditions. Data are expressed as the mean ± S.D. (n = 3). Significant differences are indicated with an asterisk at P < 0.05 and two asterisks at P < 0.01.

**Figure 17:**
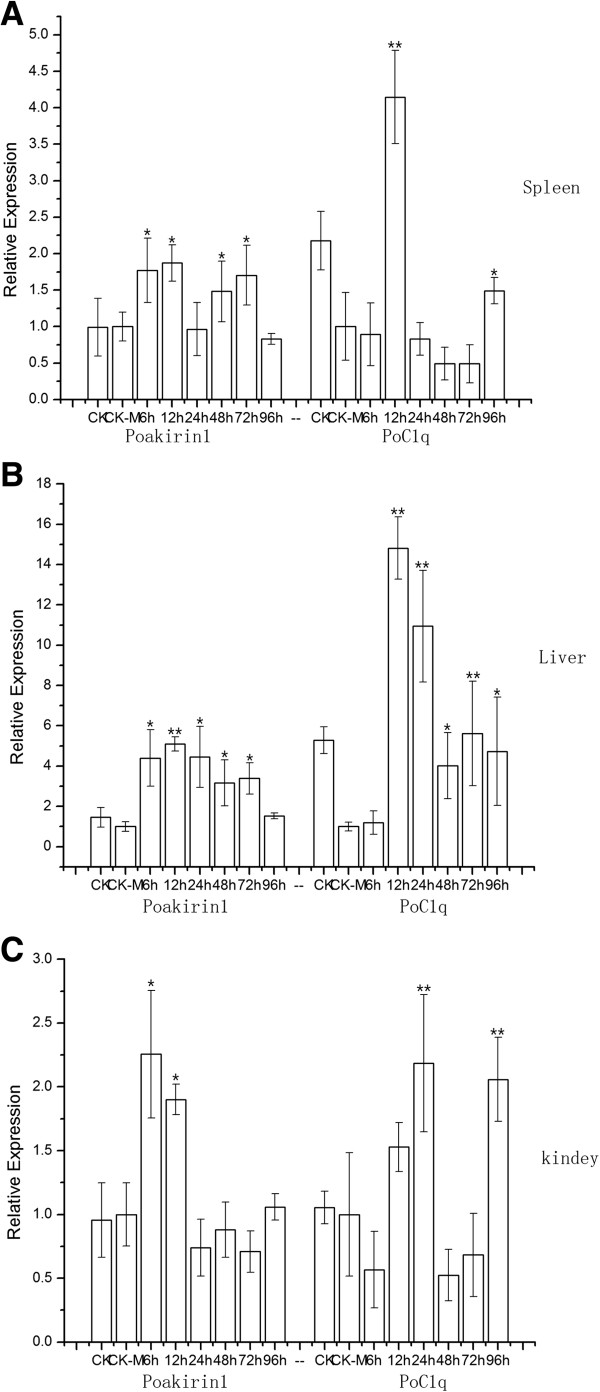
**Quantitative RT-PCR analysis of the expression levels of *PoAkirin1 *and *PoC1q *mRNA, relative to β-actin mRNA, in the spleen (A), liver (B), and kidney (C) after injection with *Vibrio anguillarum*.** Graphs show the relative expression levels of *PoAkirin1* and *PoC1q* at 6 h, 12 h, 24 h, 48 h, 72 h and 96 h post-injection. CK-M represents the tissues at 12 h after saline solution injection, and CK represents live fish under normal conditions. Data are expressed as the mean ± S.D. (n = 3). Significant differences are indicated with an asterisk at P < 0.05 and two asterisks at P < 0.01.

To determine the expression profile of *PoAkirin1*, *PoHEPN* and *PoC1q* mRNA in the challenged flounder, their mRNA levels were examined in three tissues. As shown in Figures 16 and 17, after infection by the bacteria, *PoAkirin1* expression initially increased and then decreased in the three tissues (liver, spleen and kidney); the highest expression was detected at 6–24 h. Similar to *PoAkirin1*, the *PoHEPN* expression could also be induced by bacteria, but the response time was 6 h later than that of *PoAkirin1* and the significant increment started at 12 h. *PoC1q* expression was induced at 12 and 24 h by the bacteria, and showed a relatively large increase. In the kidney and spleen, the expression levels of PoC1q were significantly higher than those of the normal control at 24 and 12 h, respectively. Significantly higher levels of *PoC1q* were also detected at 12 and 24 h in the liver (Figures 16 and 17).

## Discussion

### The HEPN domain and the sacsin protein

Spastic ataxia of Charlevoix-Saguenay (SACS) originated from the Lac-Saint-Jean region, Quebec, in Canada. The sacsin gene, the pathogenic gene of SACS, consists of nine exons, including a gigantic exon of over 12.8 kbp, which is the biggest exon in vertebrates. The gene encodes the sacsin protein, 65% certain to be located in the in the nucleus. By SMART [29] analysis, this protein has a recognizable DnaJ conserved domain, which is involved in the interaction of sacsin and Hsp70. The sacsin protein has the other three domains, including a UBQ region at the N-terminus, a HEPN domain at the C-terminus and another DnaJ region upstream of the HEPN domain.

The HEPN domain comprises 110 amino acid residues at C-terminus of the sacsin protein. In some invertebrates, bacteria and archaea, sacsin homologous proteins have this conserved domain.

### Proteins with HEPN Domains are of three main types

a) A single HEPN domain. In many types of bacterium, the protein is usually followed by nucleotidyltransferase (NT), which is often in a different reading frame, with both ORFs overlapping. Only two exceptions were found: in the genomes of *Pyrococcus furiosus* and *P. abyssi*, the HEPN protein does not exist after the NT protein.

b) Two HEPN domains, the N-terminal NT domain and C-terminal HEPN domain.

c) HEPN proteins with a variety of conserved domains. These occur mainly in fish and mammals (monkeys, rats, mice and humans). The HEPN domain is located at the C terminus of the protein, adjacent to the DnaJ domain. However, conserved HEPN domains in lower eukaryotes have not been found [30].

The function of the HEPN domain in sacsin is not clear and is difficult to predict. HEPN has an important role in stabilizing nucleotide binding in complexes formed with the DnaJ domain and may be involved in determining the specificity. In diseases caused by sacsin mutations, sacsin mutant proteins have an incomplete DnaJ and HEPN conserved domain [31]. Thus, the functions of HEPN and sacsin proteins require further study.

### C1q

Complement component C1 is composed of three subunits: Clq, C1r and C1s [32]. C1q, in C1, combined with immune complexes (IC) or other non-immune complexes, activates the classical pathway of complement activation. The C1q complement system, activated via the classic, bypass and mannose-binding lectin activation pathways, is composed of 30 types of protein. C1q has two functional units: a collagen-like region (CLR) near the N-terminus and a globular region (GR) at C-terminus. The CLR of C1q, with C1r and C1s, forms the C1 macromolecules (C1qC1r2C1s2). After the recognizing and binding to the GR through the IC, C1r and C1s are activated, thereby initiating the classical pathway. When complement is activated, a number of complement proteins can be cleaved into a variety of small cell surface fragments that are recognized by complement receptors. The primary function of the cell surface complement receptors is to promote the natural immune system to remove the foreign proteins, cell debris and microorganisms from the circulatory system [33,34].

### PoAkirin1 interacts with the N-terminus of PoHEPN

The HEPN conserved domain is predicted to locate in the 97–223 amino acids region of PoHEPN. Therefore, in the mutation experiment of PoHEPN segment deletion, we constructed three vectors comprising 1-96aa (pohepn1), 1-146aa (pohepn2), and 147-224aa (pohepn3), to verify whether PoHEPN can interact with PoAkirin1, where the binding site of PoHEPN with PoAkirin1 is, whether the interaction is associated with the conserved sequences, and whether the incomplete sequence of PoHEPN retains its binding activity. The result showed that the region of PoHEPN that binds with PoAkirin1 is located in the 1-96aa region, before the HEPN domain, and is unrelated with the HEPN domain. This suggests that the nucleoprotein PoAkirin1 has no transcriptional activation activity and must be combined with other proteins (transcription factors or other DNA-binding proteins), such as PoHEPN, which can bind DNA and initiate expression of immune-related genes as a complex. In this process, PoAkirin1 is likely to play a specific-enhancer role; however, this hypothesis should be tested by further experimentation.

### PoAkirin1 and PoHEPN expression in *E. coli* and western blotting analysis

Prokaryotic fusion protein expression vectors for PoAkirin1 and PoHEPN were constructed and the recombinant PoHEPN and PoAkirin1 were successfully expressed in *E. coli*. The recombinant proteins were verified by western blotting. The recombinant proteins will be separated and purified. These recombinant proteins will be useful for further investigation of the function of PoAkirin1, such as to verify the binding activity of PoAkirin1 with PoHEPN, changes in protein expression levels, in situ hybridization, crystal structure, and protein activity.

### Expression profiles of PoAkirin1, PoHEPN, PoC1q

To investigate the expression profile of PoAkirin1, the mRNA expressions of PoHEPN and PoC1q in different tissues from normal and *V. anguillarum* challenged fish were examined at different time points in three important immune organs: the kidney, spleen and liver. The results showed that PoAkirin1 was highly expressed in all the three tissues. The expression profile suggested that PoAkirin1 might be involved in growth, development and, especially, immunity. In most normal tissues, the expression of PoHEPN did not differ significantly. It was only slightly higher in the pituitary and skin than in other tissues. PoC1q was hardly expressed in muscle, slightly expressed in brain, gills, pituitary, skin, heart and eyes, but highly expressed in the liver, spleen, kidney and head kidney. In the liver and spleen, the expression level is significantly different compared with other tissues (p < 0.01). The expression in liver is 25 times higher than in the brain and 100 times higher than in the muscle. The reason for this tissue-specific expression may relate to their different functions. The sacsin protein has mainly been associated with nerve function [31,35]. Therefore, although it is expressed in various tissues and organs, its expression is higher in the pituitary and the skin. However, for the C1q protein, as a subunit of complement C1, its distribution is mainly in the immune tissues, where it is involved in a series of signal transductions.

The expressions of the PoAkirin1, PoHEPN, and PoC1q gene were all induced by bacterial challenge, where the expression of PoAkirin1 reached its highest point reached after 12–24 h. Interestingly, however, the responses to the bacterial challenge of PoHEPN and PoC1q were slower than that of PoAkirin1, which may indicate that PoHEPN and PoC1q are located downstream in the response to bacterial challenge, and that the change in expression of PoAkirin1 leads directly to changes in the expressions of PoHEPN and PoC1q. However, the specific relationship between the upstream and downstream proteins in this signaling pathway requires further study.

## Conclusions

In this paper, we cloned the Akirin1 homologous gene, PoAkirin1, from Japanese flounder and identified two proteins that potentially interact with PoAkirin1. The expression patterns of PoAkirin1 and the genes encoding the interacting proteins are closely associated with immunity. Bacteria can induce the expression of these genes; therefore, the function of this protein merits further investigation, particularly in the context of protecting fish populations.

## Competing interests

The authors declare that they have no competing interests.

## Authors’ contributions

S-LC obtained the project. S-LC and C-GY designed the study. C-GY, X-LW, BZ, BS, and S-SL carried out the molecular genetic studies, participated in the sequence alignment and drafted the manuscript. C-GY, X-LW, and BZ carried out the immunoassays. BS and S-SL participated in the statistical analysis. S-LC revised the manuscript. All authors read and approved the final manuscript.
